# From Mechanism-Based Retaining Glycosidase Inhibitors to Activity-Based Glycosidase Profiling

**DOI:** 10.1021/jacs.4c08840

**Published:** 2024-08-30

**Authors:** Marta Artola, Johannes M. F. G. Aerts, Gijsbert A. van der Marel, Carme Rovira, Jeroen D. C. Codée, Gideon J. Davies, Herman S. Overkleeft

**Affiliations:** # Leiden Institute of Chemistry, Leiden University, 2300 RA, Leiden, The Netherlands; & Departament de Química Inorgànica I Orgànica & IQTCUB, Universitat de Barcelona, Barcelona 08028, Spain; † Institució Catalana de Recerca i Estudis Avançats (ICREA), Barcelona 08020, Spain; $ Department of Chemistry, The University York, Heslington, York YO10 5DD, United Kingdom

## Abstract

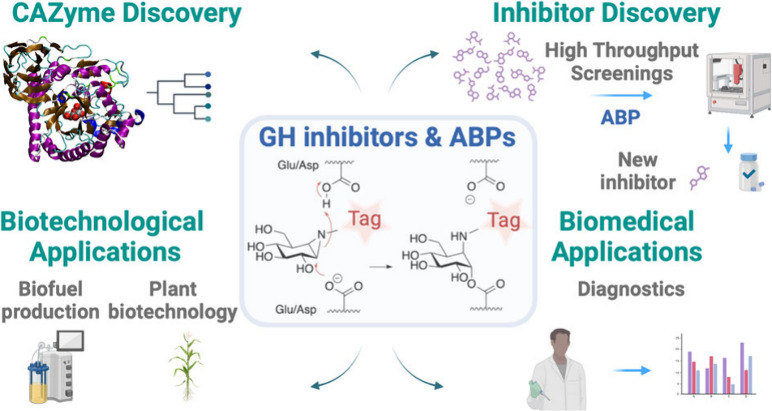

Activity-based protein profiling (ABPP) is an effective technology for the identification and functional annotation of enzymes in complex biological samples. ABP designs are normally directed to an enzyme active site nucleophile, and within the field of Carbohydrate-Active Enzymes (CAZymes), ABPP has been most successful for those enzymes that feature such a residue: retaining glycosidases (GHs). Several mechanism-based covalent and irreversible retaining GH inhibitors have emerged over the past sixty years. ABP designs based on these inhibitor chemistries appeared since the turn of the millennium, and we contributed to the field by designing a suite of retaining GH ABPs modeled on the structure and mode of action of the natural product, cyclophellitol. These ABPs enable the study of both exo- and endo-acting retaining GHs in human health and disease, for instance in genetic metabolic disorders in which retaining GHs are deficient. They are also finding increasing use in the study of GHs in gut microbiota and environmental microorganisms, both in the context of drug (de)toxification in the gut and that of biomass polysaccharide processing for future sustainable energy and chemistries. This account comprises the authors’ view on the history of mechanism-based retaining GH inhibitor design and discovery, on how these inhibitors served as blueprints for retaining GH ABP design, and on some current and future developments on how cyclophellitol-based ABPs may drive the discovery of retaining GHs and their inhibitors.

## Introduction

**Figure 1:**
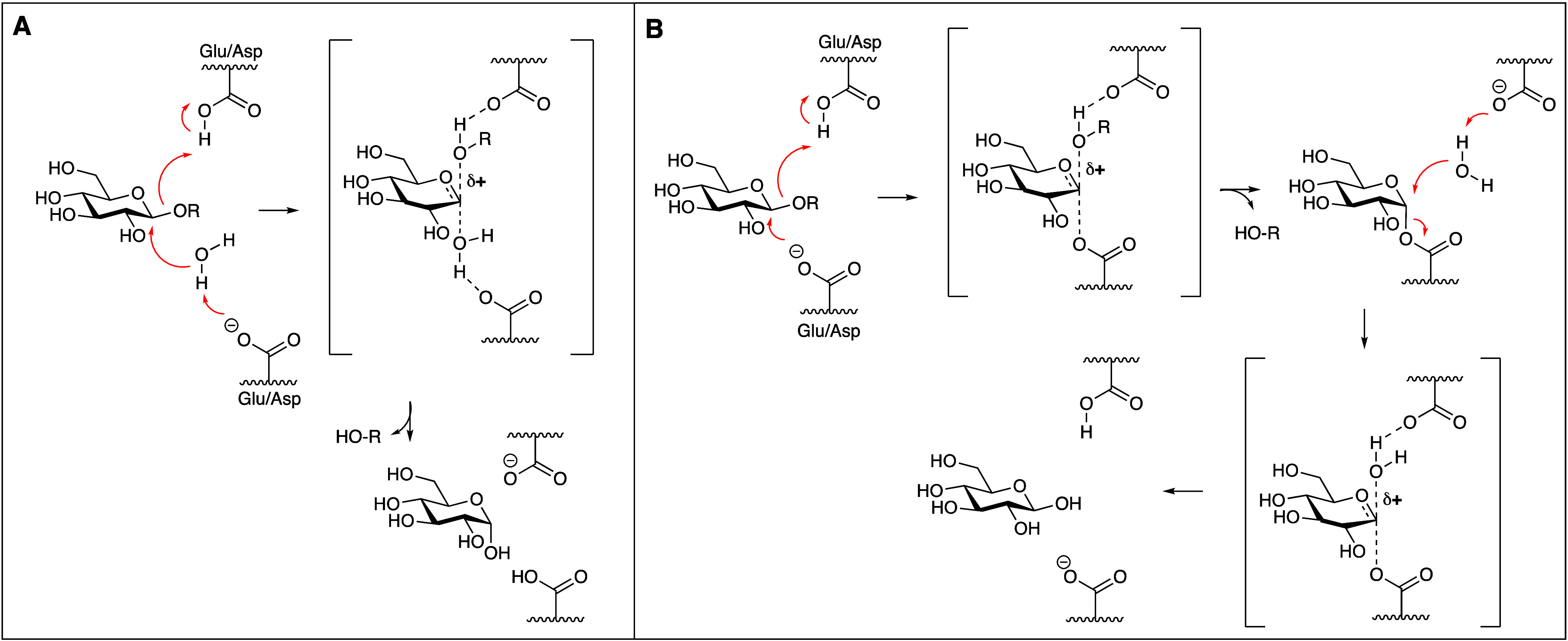
Mechanism of action of inverting β-glucosidases (**A**) and retaining β-glucosidases (**B**).

Sugars comprise the most abundant and among the structurally most diverse class of biomolecules on earth. Their structural diversity and the countless biological functions in which they partake is reflected by the enzymes that have evolved to create and break down glycans and that are collectively referred to as Carbohydrate-Active Enzymes (CAZymes). Glycosyltransferases (GTs), transglycosidases and phosphorylases catalyze the formation of glycosidic linkages, the breakdown of which is done by several enzyme families that are characterized by distinct chemistries. The largest family of glycoside-degrading enzymes are the glycoside hydrolases (glycosidases, GHs), classified in the CAZy database^1,2^ into almost two hundred families based on primary sequence (and thus predictive of structure/fold and mechanism), with two main types when looking at the stereochemical fate of the anomeric carbon at which hydrolysis takes place.^3^ These follow the mechanisms first proposed by Daniel Koshland in 1953:^4^ single displacement for inverting GHs, and double displacement for retaining GHs. Inverting GHs (illustrated in Figure 1A for an inverting β-glucosidase) catalyze substrate hydrolysis with inversion of anomeric configuration. In this process, the general acid–base residue (normally an aspartic acid or glutamic acid) residing in the inverting GH active site protonates the exocyclic acetal oxygen, thereby turning it into a good leaving group. This process coincides with deprotonation of an active site-residing water molecule, effected by an active site aspartate/glutamate, leading to overall substitution of a β-glucoside into α-glucose – thus substrate hydrolysis with net inversion of anomeric configuration. In this reaction, the substrate glucoside proceeds through an oxocarbenium ion-like transition state, with the endocyclic C-O bond developing double bond character with a partial positive charge (δ+) distributed over this C-O bond.

Retaining glycosidases (illustrated in Figure 1B for a retaining β-glucosidase) catalyze substrate hydrolysis with overall retention of anomeric configuration: a β-glucoside substrate is hydrolyzed to give β-glucose. As with inverting glucosidases, two active site carboxyl residues make up the catalytic machinery, but in contrast to the inverting glucosidase situation there is no room for a water residue within the enzyme active site upon substrate binding. Rather, the Asp/Glu residue residing at the bottom face of the substrate β-glucoside is situated closer to the substrate such that, upon protonation this residue substitutes the aglycon to form a covalent enzyme–substrate intermediate, via an oxocarbenium ion like transition state. Water then enters the enzyme active site and in a reversal of steps the glycosyl-enzyme linkage is hydrolyzed to form β-glucose, again through an oxocarbenium like transition state. Both steps proceed with inversion of configuration at the substrate anomeric carbon, and the net result is therefore retention of anomeric configuration. The existence of a covalent intermediate such as emerges during retaining β-glucosidase catalysis provides an opportunity for the design of mechanism-based inhibitors,^5^ and from there, activity-based probes. In contrast, ABP designs for inverting glycosidases are not so obvious, and for this reason, CAZyme ABPP has focused almost exclusively on studies of retaining glycosidases. The retaining mechanism is conserved over many GH families, both exo-acting (recognizing a chain-end and removing a sugar of defined length, usually a single monosaccharide but disaccharide and trisaccharide liberating exoenzymes are also known) and endo-acting (cleaving in the middle of a glycan). Most retaining glycosidases feature two Asp/Glu active site residues as general acid/base catalyst (for protonation of the aglycon) and catalytic nucleophile, respectively. Variations in general acid/base residue (His in, among others, fucoidanases^6^) and nucleophilic residue (Tyr in retaining neuraminidases,^7^ Cys in some retaining arabinofuranosidases^8^) have been identified but also these enzymes process their substrate through a covalent glycosyl-enzyme intermediate and are therefore amenable to ABPP. Notable exceptions of retaining glycosidases are hexosaminidases and chitinases that utilize substrate assisted catalysis, in which a substrate residue engages as a nucleophile^9^ and for which no suitable ABP designs have yet emerged. GH99 α-endomannanases as well utilize neighboring group participation and catalyze substrate hydrolysis through a 1,2-anhydromannose intermediate, again posing challenges for a classical ABPP approach.^10^

## Mechanism-Based Retaining Glycosidase Inhibitor Designs

**Figure 2:**
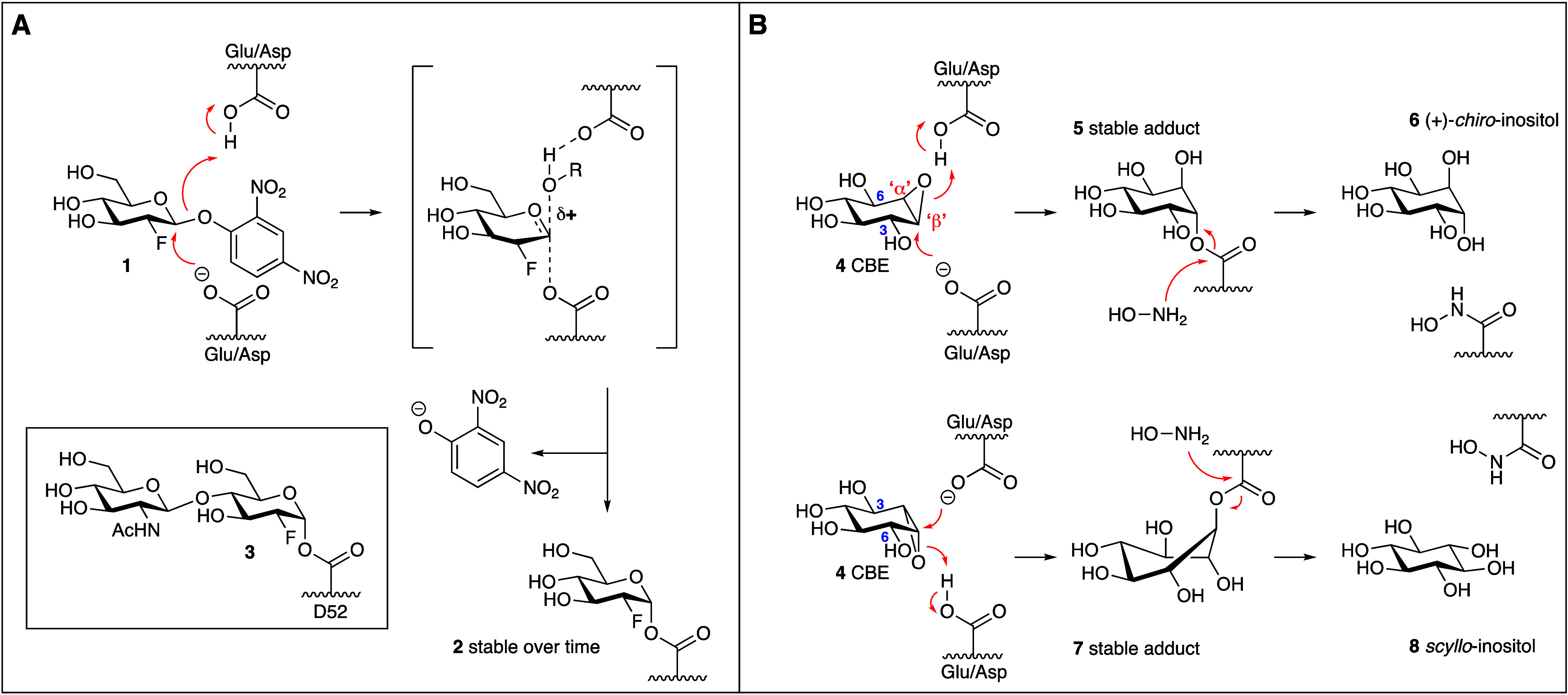
Two archetypal retaining glycosidase inhibitor designs: deoxyfluoroglycosides (**A**) and cyclitol epoxides (**B**).

Several mechanism-based, covalent and irreversible retaining GH inhibitor designs have emerged over the past decades, some of which have in later years also been adapted for ABP designs. The two most widely applied design principles are based on the two classical retaining glycosidase inactivators depicted in Figure 2A. 2-Deoxy-2-fluoroglucoside **1** was developed by Withers and co-workers in 1987 as a mechanism-based retaining β-glucosidase inhibitor.^11^ The presence of an electron-withdrawing fluorine substituent (the 2-F group in **1**) was envisaged to slow down the reaction rate through raising the energy of the oxocarbenium ion-like transition states in both steps of the retaining glycosidase mechanism (see Figure 1B). However, the inclusion of a highly reactive 2,4-dinitrophenyl selectively accelerates the first step relative to the second step. The resulting 2-deoxy-2-fluoroglycosyl-enzyme adduct **2** accumulates and depending on the enzyme can have a half-life of minutes, hours or even days, resulting in inhibition of the enzyme. The deoxyfluoro glycoside design, also termed ‘activated fluorinated glycosides’, has been used in the following years in the establishment of such nucleophiles in a range of retaining glycosidases.^12^ One seminal study^13^ demonstrates, by X-ray crystallography and mass spectrometry, that hen egg white lysozyme employs the Koshland two-step double displacement mechanism^4^ and that the active site residue, Asp52, acts as a nucleophile rather than an oxocarbenium ion-stabilizing carboxylate, forming adduct **3** (Figure 1A, insert).

Preceding the activated deoxyfluoro glycoside design is Legler’s work on conduritol B epoxide (CBE, **4**, Figure 2B).^14−17^ CBE is pseudosymmetric, and mimicks β-glucosides (Figure 2B) and α-glucosides (Figure 2A) through rotation by 180° around the axis dissecting the C1-C2 and C3-C4 bonds. This feature of CBE explains the ability of this molecule to inhibit retaining β-glucosidases and retaining α-glucosidases. Treatment of an *Aspergillus wentii* retaining β-glucosidase with **4** followed by reaction of the formed enzyme–inhibitor adduct **5** with hydroxylamine released (+)*-chiro*-inositol **6**.^16^ In contrast, treatment of rabbit intestine sucrase-isomaltase, a retaining α-glucosidase, with CBE **4** followed by reaction of the adduct with hydroxylamine, returned *scyllo*-inositol **8**.^17^ These results strongly suggested enzyme-mediated, regioselective opening of the epoxides at the carbon atom assuming, within the active site of the respective enzymes, the position of the anomeric carbon of the β- and α-glucoside substrates, respectively, providing supporting evidence for the two-step double displacement mechanism proposed by Koshland.^4^

**Figure 3:**
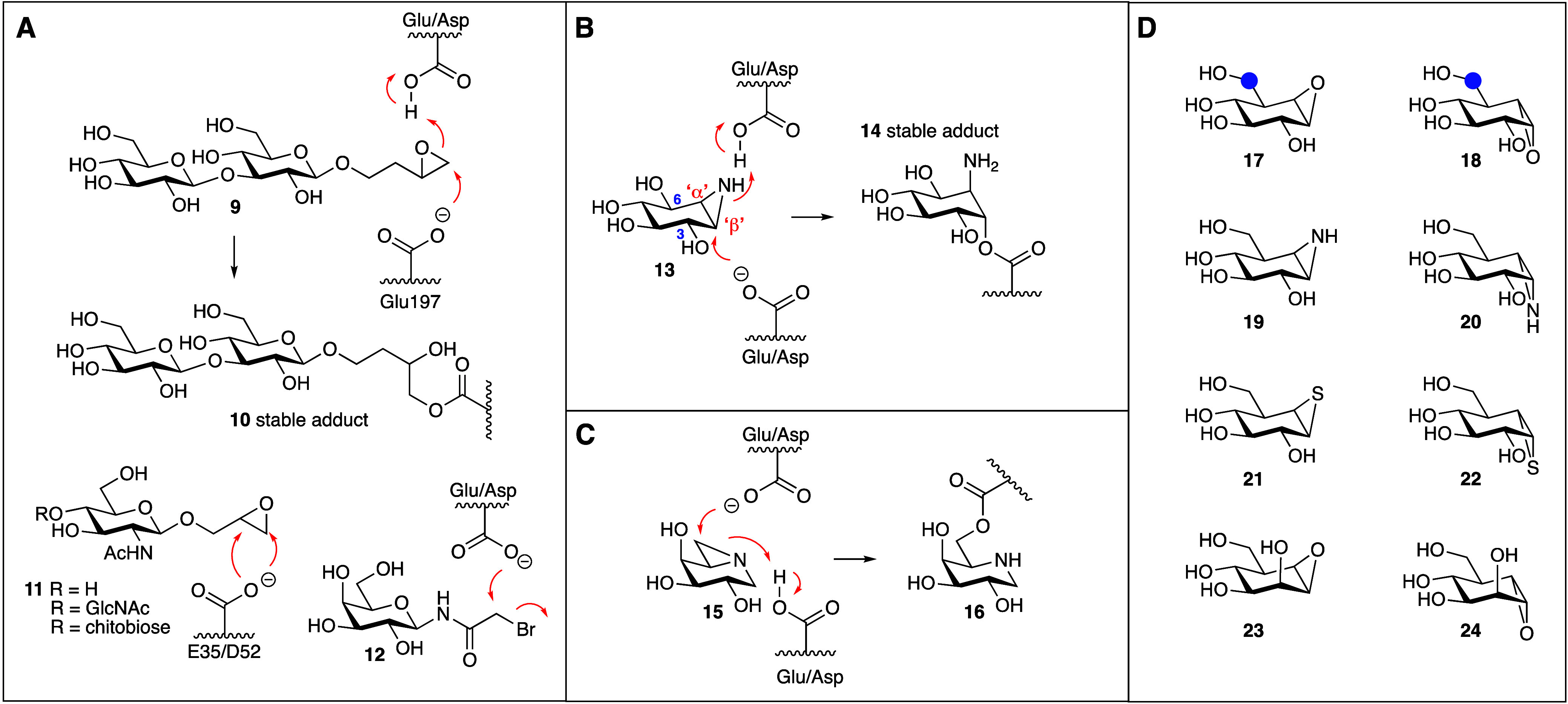
Glycomimetic epoxides, aziridines and episulfides as mechanism-based, covalent and irreversible retaining glycosidase inhibitors.

CBE (**4**) is a member of a series of carbohydrate-mimetic epoxides and aziridines that have been used for many decades as GH inactivators to study the mechanism of (retaining) GHs. Besides cyclitol-fused epoxides, also linear, glycosylated epoxy alcohols have contributed to unearthing retaining GH active site nucleophiles.^18,19^ The example shown in Figure 3A pertains an X-ray study revealing that both diastereomeric epoxides from a mixture of 3,4-epoxybutyl-β-cellobioside **9** reacted within the *Fusarium oxysporum* cellulase, EG I, to form a covalent ester bond with the active site nucleophile (E197).^20^ Such epoxy-alkylglycosides have been applied to inactivate retaining glycosidases since 1969, when Thomas^21^ used GlcNAc-epoxides **11** for the irreversible inactivation of hen egg white lysozyme (HEWL) in a study aimed to gather evidence for the Koshland two-step mechanism invoking an active site nucleophile employed by retaining glycosidases.^4^ Although the mode of action (reaction of either of the epoxide carbons with either of the active site carboxylates E35 or D52) was not revealed in these studies, HEWL was shown to be inhibited irreversibly. Perhaps because linear epoxides such as **9** are rather weak GH inhibitors, and because later studies revealed they may alkylate both nucleophile and general acid base residues at the same time,^22^ these compounds have not been used as a starting point for glycosidase ABP design, even though grafting a reporter moiety on either terminus of the molecule appears a relatively simple manner. It may also be that, when in 1999 the concept of ABPP emerged,^23^ better design blueprints had already been identified. Besides epoxides, haloacetyl aminoglycosides have been used as mechanism-based retaining GH inactivators since the early seventies of the last century as well. *Escherichia coli* retaining β-galactosidase inactivator **12** comprises the first example of this compound class,^24^ which has been adapted in later years in ABP designs.

Returning to the theme of cyclic epoxides and aziridines, Caron and Withers in 1989 reported conduritol B aziridine **13** as a mechanism-based inactivator of both almond β-glucosidase and yeast α-glucosidase, thus emulating the activity profile also exhibited by CBE **4**.^25^ This study was preceded by one year by the design, by Tong and Ganem, of the coffee bean α-galactosidase inactivator **15**.^26^ Although the proposed mechanism of inactivation (the formation of **16**) as shown in Figure 3C was not proven, the compound blocked the enzyme in an irreversible manner and proved to be selective over yeast α-glucosidase and jack bean α-mannosidase. In their work on conduritol B aziridine **13**, Caron and Withers predicted^25^ that breaking the pseudosymmetry in CBE (or conduritol B aziridine) by inserting an extra methylene in the C3-OH or C6-OH bonds would yield compounds both more potent and more selective for retaining α and β-glucosidases, respectively. The discovery,^27^ one year later, by Umezawa and co-workers, of the natural product, cyclophellitol (**17**) proved this idea to be valid. Cyclophellitol, the CBE analogue having the added methylene (the blue bulb) inserted in the C6-OH bond, is a highly potent and selective (also with respect to retaining α-glucosidases) retaining β-glucosidase inhibitor, and X-ray studies by Gloster, Madsen and Davies revealed that the mode of action is indeed by active site nucleophile modification, in particular the *Thermotoga maritima* retaining β-glucosidase (*Tm*GH1) active site nucleophile, Glu351.^28^ Shortly following the discovery of cyclophellitol, 1,6-epi-cyclophellitol (**18**, with a methylene now inserted in the indicated C3-OH bond in CBE) was synthesized and shown to be an effective and selective retaining α-glucosidase inactivator.^29^ As part of these studies also the corresponding aziridines **19** and **20** were synthesized and shown to be potent retaining β-glucosidase (**19**) and retaining α-glucosidase (**20**) inactivators. Episulfides **21** and **22** in contrast proved much less active, while configurational isosters such as β- and α-mannose-configured cyclophellitols **23** and **24** were put forward as potential inactivators of the corresponding retaining GHs.^30^

## The Advent of Activity-Based Retaining Glycosidase Profiling

**Figure 4:**
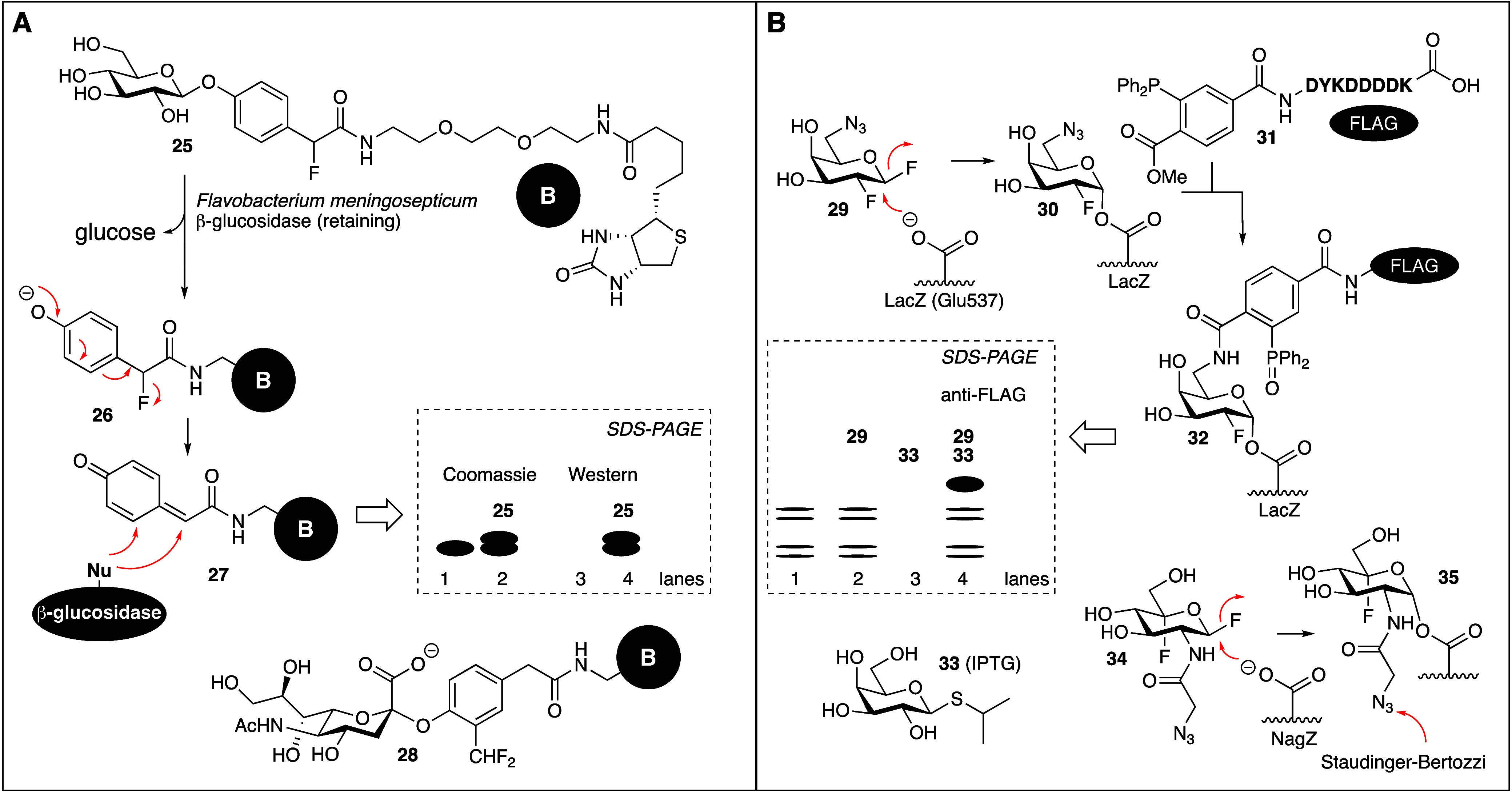
Latent quinone methides (A) and activated fluorinated glycosides (B) as activity-based (retaining) GH probes. For the original images of the SDS-PAGE gels that are shown here and in the remainder of this account, please see the papers referred to in the text.

Cravatt and co-workers introduced the concept of ABPP 25 years ago^23^ when they used a biotin-fluorophosphonate construct (termed FP-biotin) to capture and identify serine hydrolases from complex biological samples. In 2000, Bogyo and co-workers demonstrated the generality of ABPP by using tagged peptide epoxysuccinates to capture cysteine proteases of the cathepsin family.^31^ Mechanism-based, covalent and irreversible retaining GH inhibitors had been known for several decades at that time, and it is therefore no surprise that the first reports on retaining GH ABPs followed shortly after these two foundational ABPP studies on serine hydrolases and cysteine proteases. The first activity-based GH probe design (2002) was not rooted in the above-described retaining GH inhibitors but in altogether different chemistries: the GH-catalyzed generation of tagged, reactive electrophiles. Inspired by the strategy^32^ of Wong and Lerner for selecting, from large pools, catalytic antibodies with glycosidase activity, Lo and co-workers designed β-glucosidase substrate **25** (Figure 4A) containing a latent quinone methide.^33^ Exposure of **25** to recombinant *Flavobacterium meningospectum* β-glucosidase generated phenolate **26** which after fluoride expulsion led to *in situ* formation of quinone methide **27**. The test enzyme used here is a retaining GH, but the strategy should work as well for an inverting one: compound **27** is a reactive electrophile designed to react with any nucleophile within or nearby the GH active site to form a covalent and irreversible adduct, thereby attaching a biotin moiety to the protein. Upon SDS-PAGE of the denatured protein mixture from the experiment in which the test GH was treated with **25**, Coomassie staining then revealed the emergence of higher molecular weight protein bands (shown are graphic representations of the original gels; for this and the ensuing examples, the reader is directed to the original papers), suggesting that reaction with one or multiple quinone methides have occurred (lane 2). The same image is returned in a streptavidin–biotin Western blot (lane 4), demonstrating that the **25**-treated GH, but not the nontreated one (lane 3) has acquired biotin residues. The authors expanded their strategy to labeling, with the latent quinone methide **28** (yielding a more reactive electrophile compared to **25**), of recombinant *Athrobacter ureafaciens* neuraminidase,^34^ but had not progressed to ABPP in cell extracts or more complex biological samples at that time. Withers and co-workers observed that such experiments may lead to diffusion from the active site of the generated quinone methide to indiscriminately label nearby proteins and capitalized on this idea by staining cells in a GH-activity-dependent manner.^35^

Vocadlo and Bertozzi were the first to capitalize on the activated fluorinated glycoside design in their development of an activity-based retaining GH probe targeting the *Escherichia coli* retaining β-galactosidase, LacZ (Figure 4B).^36^ In their strategy they combined bioorthogonal chemistry with mechanism-based retaining GH inhibition, and designed 1,2,6-trideoxy-1,2-difluoro-6-azido-β-galactoside **29** based on previous findings^37^ that the 6-OH analogue covalently and irreversibly modifies the LacZ nucleophile, Glu537. They then cultured *Escherichia coli* in the absence or presence of the LacZ inducing agent, isopropyl-β-D-thiogalactoside **33**, and treated extracts of these cells with **29**, and then with the Staudinger-Bertozzi phosphine **31** carrying a FLAG tag. Besides some background labeling a major protein band in the ensuing anti-FLAG Western blot of SDS-PAGE separated proteins was returned (lane 4) only when cells were treated with **33**, then **29** and finally the bioorthogonal reagent, **31**. Two-step ABP **29** proved in-class active toward a few retaining β-galactosidases and modified almond β-glucosidase as well. The strategy was expanded^38^ a few years later using the bioorthogonal activated fluorinated GlcNAc analogue **34** to label, in *Pseudomonas aeruginosa* extracts, the retaining β-glucosaminidase, NagZ (formation and bioorthogonal detection of adduct **35**) and influenza neuraminidases.^39^ As well, Hekmat and Withers expanded the strategy to capture and identify endoglycosidases in their work on the identification of a new xylanase expressed by *Cellulomonas fimi*.^40^ Following these studies, suites of GH ABPs composed of latent quinone methides, activated deoxyfluoro glycosides and haloacetyl aminoglycosides were used by Wright and co-workers^41^ to study cellulose-degrading enzymes produced by *Clostridium thermocellum*. Many carbohydrate-active proteins from various cellulosomes were captured in this manner, but the number of off-targets and promiscuous amino acid side chain labeling made data interpretation somewhat arduous.

## Cyclophellitol Aziridine Isosters in Activity-Based Retaining Exoglycosidase Profiling

In the year following the conception of ABPP when we started work on the design of GH ABPs, none of the ABP designs in Figure 4 had been reported. To us, the activated deoxyfluoro glycosides and the cyclophellitols/cyclophellitol aziridines appeared most suited for retaining GH ABPP. They present an ‘anomeric’ electrophilic carbon (instead on one a few atoms removed) to the enzyme active site nucleophile, which we thought may impact activity and active side nucleophile-selectivity. Moreover, glycoside/glycomimetic configuration in both designs appeared to match well with retaining GH selectivity. Of the two design blueprints, we considered the cyclophellitol one the most attractive. In contrast to activated deoxyfluoro glycosides,^42^ enzyme reactivation by hydrolysis of reacted epoxides had not been shown, and substitution of the epoxide for an aziridine would create room for installing a reporter moiety without the need to sacrifice another of the hydroxyl functionalities. We felt this may be important for initial enzyme active site binding and noted that in most activated deoxyfluoro glycoside designs OH-2 is already sacrificed for fluorine. The downside of this choice we thought would be the lengthier and more complicated synthesis schemes required for the preparation of cyclophellitol-based ABPs as opposed to activated, fluorinated glycoside ones. This turned out to be true and it was only after Robert Madsen published^43^ his total synthesis of cyclophellitol (**17**) that we were able to generate our first retaining GH ABPs.

**Figure 5:**
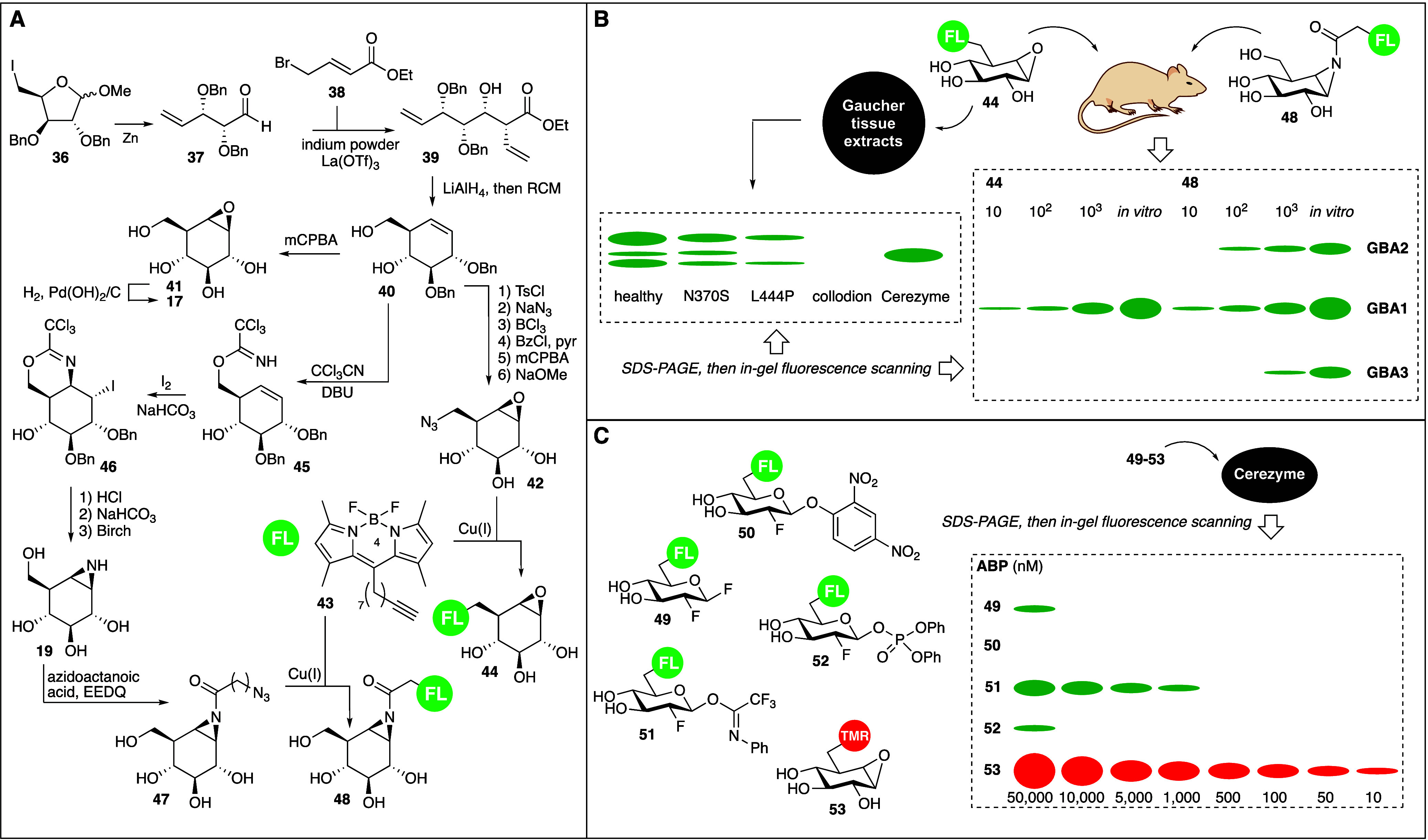
Retaining GH ABPs based on cyclophellitol and cyclophellitol aziridine. A) Adaptation of the Madsen cyclophellitol synthesis to give cyclophellitol and cyclophellitol aziridine ABPs **44** and **48**. B) Comparative ABPP of the human retaining β-glucosidases, GBA1, GBA2 and GBA3. C) Head-to-head comparison of activated fluorinated glucosides **49**-**52** and cyclophellitol **53** as GBA1 ABPs.

The Madsen route proved reliable, scalable, and adaptable. A key step in Madsen’s synthesis (Figure 5A) is the lanthanum triflate-catalyzed, indium-mediated Barbier addition of ethyl-4-bromocrotonate **38** to xylose-derived 4-pentenal **37**. Reduction of the methyl ester in the resultant 1,7-diene **39** and ring-closing metathesis gives cyclohexene **40** with the four chiral carbon centers emulating the glucopyranose configuration. The homoallylic primary alcohol in **40** allows for stereoselective epoxidation of the double bond to yield **41**, and palladium-catalyzed hydrogenolysis of the two benzyl ethers then gives cyclophellitol (**17**).

For our first retaining GH ABP design,^44^ we took advantage of partially protected cyclohexene **40**, the primary alcohol of which we transformed into the corresponding azide by first selective tosylation and then azide displacement of the resultant tosylate.^45^ Protecting group manipulation and epoxidation of the double bond then gave two-step ABP **42**, and copper(I)-catalyzed azide-alkyne click ligation Bodipy-FL-tagged direct ABP **43**. This probe (which we referred to as MDW933) and its red-fluorescent analogue **53** (Figure 5C, MDW941) we then applied in our first forays into activity-based retaining GH profiling studies. Cyclophellitol **44** proved to be highly active and very selective in labeling the human lysosomal retaining β-glucosidase, glucosylceramide (glucocerebroside, GBA1).^44^ Genetic deficiency in GBA1 is at the basis of the lysosomal storage disorder, Gaucher disease, and a variety of mutations in the gene encoding for GBA1 can lead to various disease states ranging from mild to severe. Mutations in the *GBA1* gene impact ER quality control, rather than catalytic activity, and disease states therefore correlate with the number of active enzymes within lysosomes. This can be quantified by ABPP, and this is what we have done with ABP **44**: we took extracts of macrophages from individuals carrying different GBA1 mutations, treated these at pH 5 with our probe, resolved the protein content by SDS-PAGE and scanned the wet gel slabs for in-gel fluorescence (Figure 5B). Compared to the sample derived from a healthy individual, the one from the N370S mutant returned a slightly weaker signal (GBA1 comes in several glycoforms leading to separated signals). The L444P mutant yielded a much weaker signal while the RECNCI collodion sample gave no signal at all. This pattern coincides with the severeness of disease, ranging from mild (N370S) to severe (L444P) to lethal (collodion). The right lane comprises a sample of recombinant GBA1 (Cerezyme) used in the clinical treatment of Gaucher disease as enzyme replacement therapy.

Our publication,^44^ in 2010, on cyclophellitol ABPs **44** and **53** was accompanied by an editorial entitled ‘getting lucky in the lysosome’.^46^ It is fortuitous that GBA1, in contrast to most other exoglycosidases, allows for substantial modification of the carbohydrate (mimetic) core. But there was no luck involved in our design. We had already established that 6-O-alkyl-deoxynojirimycins inhibit GBA1, the natural substrate of which is glucosylceramide (so, a glucosylated lipid) almost equally potently as its N- or C1-alkyl counterparts.^47^ We also knew that substitution of the epoxide for an aziridine yields equally potent retaining GH inhibitors and anticipated that grafting a fluorophore onto the aziridine nitrogen would yield ABPs with the bulky reporter moiety in the direction of a substrate aglycon, a direction that should be generally tolerant of large groups. Many exoglycosidases are particular to the nature (configuration, substitution pattern) of their substrate monosaccharide but considerably less so to the aglycon and we felt that such a design would yield a general template for retaining exo-GH ABPs not offered by the other design blueprints. To test this hypothesis, we made N-acyl-cyclophellitol aziridine **48**, for which we again turned to the Madsen intermediate **40**.^43,45^ Key step in this synthesis comprises iodocyclization of imidate **45** to deliver the nitrogen to the top face of the alkene. Hydrolysis of the resultant iminal was then followed by intramolecular iodine displacement to give, after Birch reduction of the two benzyl ethers, cyclophellitol aziridine **19**. N-Acylation (in most following studies we opted for N-alkylation) and click ligation then gave ABP **48**. This probe indeed proved reactive to all three retaining β-glucosidases expressed constitutively in mice and man: GBA1, GBA2 and GBA3. This we demonstrated (Figure 5B) in a comparative study,^48^ in which we treated mice intravenously with varying concentrations of either the GBA1-selective ABP **44** or with ABP **48**, after which the animals were sacrificed, their tissues harvested, homogenized, and the resultant protein mixtures resolved by SDS-PAGE. Scanning of the resultant gel obtained from liver homogenates shows one major, concentration-dependent band for animals treated with **44**, and three concentration-dependent bands for **48**-treated mice. These bands correspond with the molecular weight of GBA3 (lowest), GBA1 (middle) and GBA2 (highest) and substitution of the fluorophore in **48** for a biotin indeed allowed for identification of GBA1, GBA2 and GBA3 by chemical proteomics (pull down of biotinylated proteins with streptavidin-coated magnetic beads, then on-bead trypsin digestion and LC-MSMS analysis of the resultant trypsin digest peptides). Finally on our first ventures into retaining GH ABPP, we directly compared cyclophellitol ABPs with their activated deoxyfluoro glycoside counterparts.^49^ The latter design does not allow grafting a reporter in the aglycon direction and therefore relies on bioorthogonal chemistries, thus on two-step ABPP approaches, excepting situations where carbohydrate (mimetic) core modification with a fluorophore or biotin is tolerated. Such as with GBA1, which allowed us to compare the labeling efficiency (concentration at which labeling of recombinant GBA1 when resolved by SDS-PAGE is still detected) of the series of activated fluorinated glucosides **49**-**52** with red fluorescent cyclophellitol **53**. ABP **53** clearly emerges as the most potent ABP from these studies. As well, the nature of the activation of fluorinated glycosides can have a major impact on potency, with the Biao Yu trifluoroimidate **51**(50) being the clear winner in this respect, and the 2,4-dinitrophenylglucoside in these studies surprisingly inactive. We did this comparison for retaining β-glucosidases only, both for direct ABPP as shown and (using **42** and the corresponding 6-azido-2-fluoroglucosides) in two-step bioorthogonal ABPP.^51^ We therefore do not wish to claim the cyclophellitol (aziridine) design blueprint is the superior one in all instances. But neither did we turn to other designs in our subsequent studies.

The Madsen cyclophellitol synthesis scheme proved not only effective for the synthesis of modified cyclophellitols and cyclophellitol aziridines, but also for the synthesis of configurational isosters. Llebaria and co-workers reported that, besides partaking in a Barbier allylation, pentenal **37** can undergo an asymmetric aldol condensation with an Evans-templated acrylate.^52,53^ This led to a precursor 1,7-diene resembling **39** but that yields, upon further processing rather like the Madsen synthesis, the β-*galacto*-configured cyclophellitol aziridine. Adaptation of either of these routes, combined with a separate route of synthesis we developed^54^ of a fully orthogonally protected cyclohexene analogue of **40**, allowed us to synthesize an array of cyclophellitol isosteres (α-glucose,^55^ α/β-mannose,^56,57^ α-fucose,^58^ α/β-galactose,^59,60^ α-L-iduronic acid,^61^ β-glucuronic acid^62^) that all proved in-class selective for their corresponding retaining GHs, some more active than others, some also more selective than others, but with the exception of the α-L-iduronic acid^61^ probe (which detects recombinant enzyme), all able to identify their target GHs in cell extracts.

## Cyclophellitol ABPs Act as Transition State Analogues in Respect of Conformation

**Figure 6:**
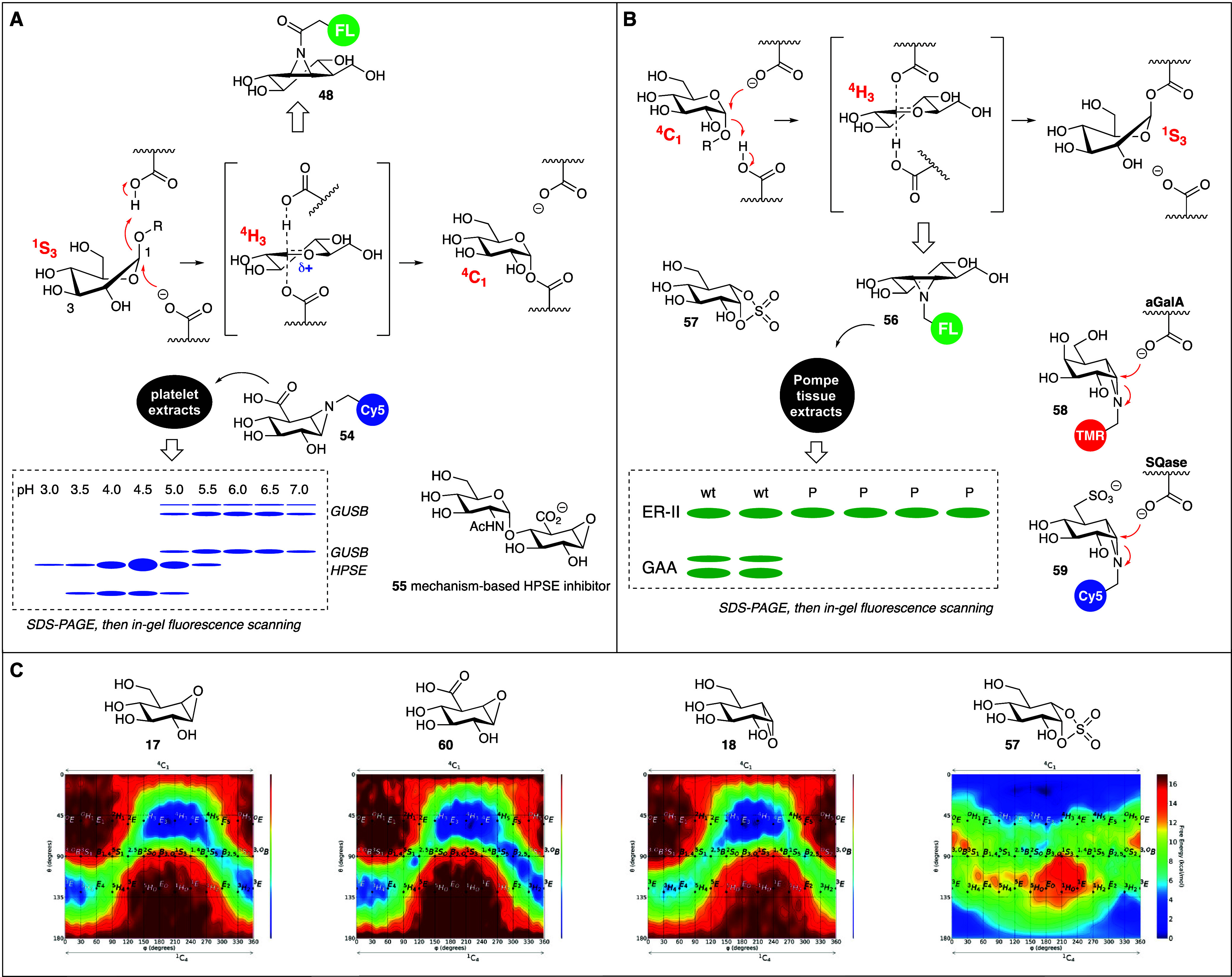
A, B) Reaction coordinates by which retaining β-glucosidases (A) and retaining α-glucosidases (B) process their substrates. C) Computed free energy landscapes (FELs) of selected compounds.

GH-catalyzed hydrolysis of glycosidic bonds proceeds through transition states having a considerable oxocarbenium ion character. Michael Sinnott predicted the emergence of chair- or boat-like transition states to accommodate the developing sp^2^-character around the C-O oxocarbenium bond during GH catalysis.^3^ Building on this, and supported by experimental and computational^63^ evidence, Davies, Rovira and Planas in their 2011 *Account* entitled ‘conformational analysis of the reaction coordinate of glycosidases’ posed that GHs (inverting and retaining alike) would distort their substrates away from their lowest energy conformation in a predictable manner to allow the formation of such half-chair or boat-like transition states.^64^ According to this evaluation, retaining β-glucosidases in general follow the ^1^S_3_-^4^H_3_-^4^C_1_ reaction itinerary as depicted in Figure 6A.

In this pathway, the substrate β-glucoside adopts a ^1^S_3_ skew boat conformation in the initial Michaelis complex such that the leaving group (aglycon) is positioned axially, allowing the formation of an endocyclic C=O double bond and thus the transition state oxocarbenium ion. This is a short-lived species, whose ^4^H_3_ half-chair conformation is then trapped by the active site nucleophile to complete the first half of the catalytic cycle, with the substrate glucoside now α-linked within the enzyme active site and having adopted a ^4^C_1_ chair conformation. It is this latter conformation (^4^C_1_) that is revealed in the structure of the *Tm*GH1 retaining β-glucosidase bound to cyclophellitol (**17**), and mechanism-based inhibitors can thus assist in determining substrate processing pathways, just as (as is put forward in the aforementioned *Account*, and before that also by Andrea Vasella^65^) reaction itineraries may assist in the design of GH-selective inhibitors. As evidenced by their computed^63^ free energy landscapes, cyclophellitol (**17**), just like cyclophellitol aziridines **19** and **48**, adopts a lowest energy ^4^H_3_ conformation. In this conformation, the epoxide/aziridine heteroatom is ideally placed for general acid–base protonation, and the ‘anomeric’ carbon for subsequent attack by the active site nucleophile. A structure of an unreacted cyclophellitol derivative within the *Tm*GH1 active site reveals this transition state conformation^66^ and together with the reacted one reveals part of the reaction itinerary shown in Figure 6A, and this conformational positioning of the electrophile for nucleophilic displacement may explain why cyclophellitol-based ABPs inhibit retaining β-glucosidases so well. It also points the way for the development of ABPs targeting retaining GHs that process their substrate through a similar set of reaction coordinates but that take on glycans other than β-glucosides. Among others, several retaining β-galactosidase and retaining β-glucuronidase families follow the ^1^S_3_-^4^H_3_-^4^H_1_ itinerary and are readily trapped by the corresponding β-*galacto*^60^ and β-*glucurono*-cyclophellitol aziridines.^62^ The latter is demonstrated in Figure 6A, showing that ABP **54** captures from platelet extract the human β-exoglucuronidase, GUSB (present in several isoforms) and surprisingly also the endoglucuronidase, heparanase (HPSE). To date this is the only retaining endoglycosidase we found to react with a monosaccharidic cyclophellitol aziridine and this finding demonstrates the power of ABPP: one may design a probe to react with a certain enzyme (family), but the unbiased nature of the technology allows for detection (through biotin-ABPs and by chemical proteomics) also of its off-targets. These may be of interest by themselves, and we realized that HPSE has been seen as a potential antitumor target for many years. It is upregulated in, and excreted by, many metastatic cancers and is thought to facilitate metastasis by degradation of the extracellular matrix (of which heparan sulfate proteoglycans make up a major component), facilitating metastatic dissemination and releasing mitogens that drive cellular proliferation. Four competitive HPSE inhibitors have undergone clinical trials, but none have reached the clinic yet. These inhibitors are all large, mostly heterogeneous and highly negatively charged to match the substrate and the extensive active site pocket, a match that cannot be met with small molecule competitive HPSE inhibitors. Mechanism-based inhibitors can overcome weak initial affinities by forming a covalent bond – a strategy that for instance has met with success in the clinical development of proteasome inhibitors. With this in mind, we prepared α-1,4-GlcNAc-*glucuronic* cyclophellitol **55** and showed this to be at least equally potent as the current best-in-class (large, heterogeneous, strongly anionic) competitive HPSE inhibitor in blocking cancer metastasis in three *in vivo* tumor models.^67^

Retaining α-glucosidases often process their substrates through an ^1^C_4_-^4^H_3_-^1^S_3_ itinerary (Figure 6B), thus the opposite pathway to that employed by retaining β-glucosidases and sharing the half-chair oxocarbenium ion transition state.^64^ The lowest energy conformation adopted by 1,6-*epi*cyclophellitol aziridine **56** is ^4^H_3_ as well and fits therefore well within the enzyme active site but now (compared to the situation depicted in Figure 6A for cyclophellitol aziridine **48**) with the aziridine pointing downward for nucleophilic displacement from the top face. ABP **56** is an effective label of lysosomal α-glucosidase (GAA) and ER α-glucosidase II (ER-II), the two retaining α-glucosidases encoded in the human genome.^55^ Deficiency in GAA is at the basis of the lysosomal glycogen storage disorder, Pompe disease and the lack of GAA, compared to ER-II, in Pompe patients (P) is revealed by comparative ABPP using **56** (Figure 6B). Retaining α-glucosidases bind their substrate in ^4^C_1_ conformation in the initial Michaelis complex (compare the ^1^S_3_-conformer in retaining β-glucosidases), which allows the design of a new and selective class of mechanism-based inhibitors. We noted some cross-reactivity of ABP **56** toward retaining β-glucosidases. This is not unexpected given that both transition states in Figures 6A and 6B are alike, and the close analogue conduritol B aziridine **13** inhibits (Figure 3B) both retaining α-and β-glucosidases. 1,6-*Epi*-cyclophellitol cyclosulfate **57** in turn emulates the ^4^C_1_ Michaelis complex conformation. This compound turned out to be a highly potent, and highly selective, inhibitor of GAA and ER-II in a study^68^ demonstrating that evaluation of reaction itineraries can indeed assist in the rational design of GH inhibitors. The ^1^C_4_-^4^H_3_-^1^S_3_ itinerary is also used by several retaining α-galactosidases, which are trapped by ABP **58**, and sulfoquinovosidases (SQases, trapped by **59**).^69^

## Activity-Based Profiling of Retaining GHs Involved in Biomass Polysaccharide Turnover

Sulfoquinovosyl diacylglycerol (SQDG) is produced by plants, algae and cyanobacteria and constitutes one of the major natural organosulfur species. The annual production of SQDG is speculated to be on a scale commensurate of the sulfur-containing amino acids cysteine and methionine. SQDG degradation is therefore an important process in the natural organosulfur cycle and control over this process may impact sulfur nutrition and the carbon cycle. GH31 sulfoquinovosidases, enzymes that hydrolyze SQDG and sulfoquinovosylglycerol, are retaining GHs that can be labeled by appropriately configured cyclophellitol aziridines such as **59** (Figure 6B). Using ABP **59** we demonstrated that SQases are expressed, by both *Escherichia coli* and *Pseudomonas putida*, when exposed to sulfoquinovose but not in the absence thereof.^69^ We also showed that, once expressed, these SQases have a relatively long lifetime and remain active for hours after their encoding mRNA has disappeared.

**Figure 7:**
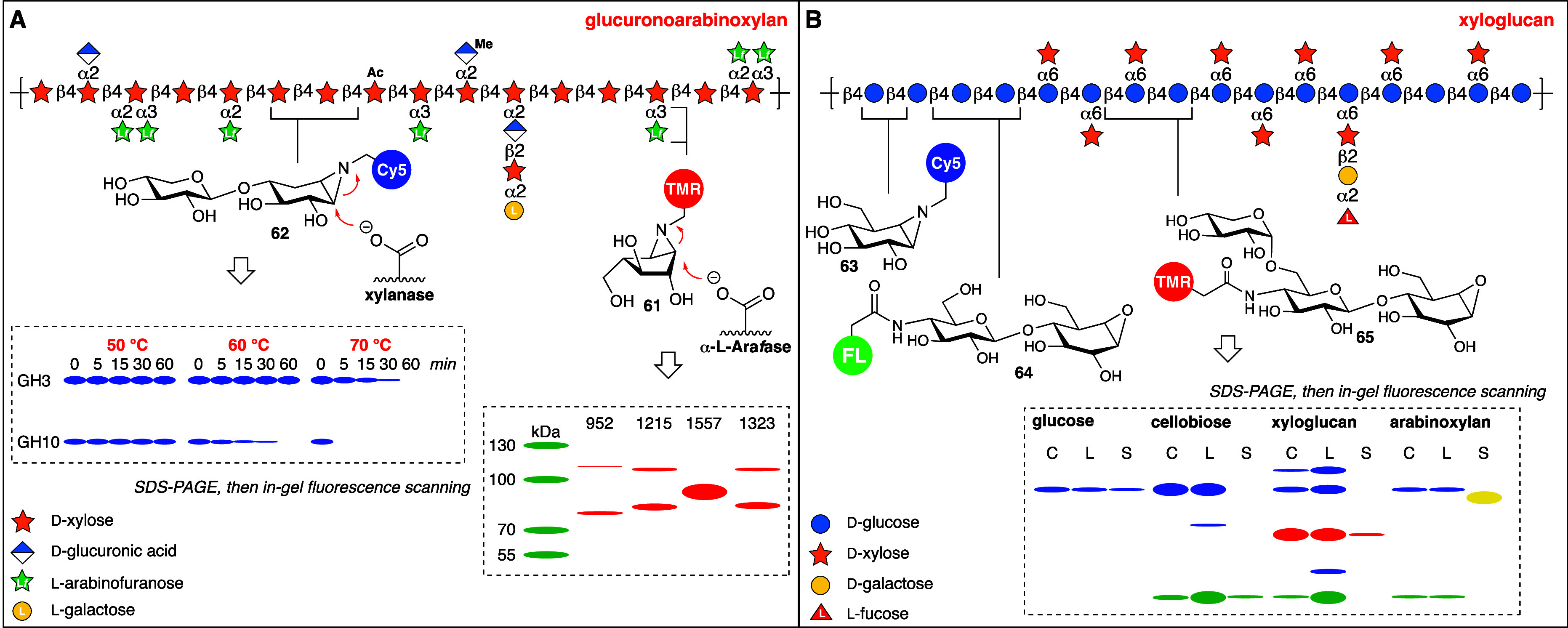
A), Activity-based secretomes profiling of glucuronoarabinoxylan-degrading retaining arabinofuranosidases and xylanases. B) Xyloglucan-degrading retaining exoglucosidases, cellulases and xyloglucanases. Shown in the gel are samples of intact cells (C), cell extracts (L) and supernatant/secretomes (S) with ABPs **63**-**65**.

The Wright^40^ laboratory has used suites of GH ABPs in their studies on microbial biomass polysaccharide degradation. In line with the SQase trapping work, we felt that our cyclophellitol design blueprint would be of use in this area as well. Some of our exploits in this direction are presented in Figure 7 and pertain the study of retaining exo- and endoglycosidases in bacterial and fungal secretions of proteins (termed secretomes) when grown on specific glycan sources. In one experiment, we treated various basidiomycete strains grown on α-L-arabinofuranoside-containing polysaccharides with α-L-arabinofuranose-configured aziridine **61**.^70^ Fluorescence scanning of the resultant SDS-PAGE gels rapidly identifies proteins that have reacted with **61** and that by this virtue may also be retaining GHs responsible for cleavage of the respective glycosidic bond, and one may for instance select strain 1557 (one major activity) for further studies.

Xylobiose-configured aziridine **62** comprises the first retaining endoglycosidase ABP we designed and emulates part of the backbone structure of the major biomass polysaccharide, glucuronoarabinoxylan.^71^ Treatment of secretomes of *Aspergillus niger* grown on this polysaccharide with **62** yielded two fluorescent bands (Figure 7A) that were revealed by chemical proteomics (using the biotin counterpart of **62**) as a GH10 xylanase (the lower band) and a GH3 exoxylosidase (the upper band). The latter likely first removes the nonreducing β-xyloside to be then confronted by a β-xylose-configured cyclophellitol aziridine, with which it then reacts to form a covalent and irreversible adduct. Beyond usage of **62** in the discovery of xylan-processing enzymes it can also be applied to study their resilience toward harsh conditions that may be required in their (industrial) biotechnological application: pH, salt concentrations and as illustrated in Figure 7A, temperature. Both GH3 and GH10 proved to retain activity after exposure to 50 °C for up to two hours (shown in the graphic representation of the original gel is up to one hour). GH3 (the exoxylosidase) retained full activity also at 60 °C and partial activity at 70 °C, at which temperature GH10 (the xylanase) was completely inactivated already after 5 min. One lesson we learned from these studies is that, in contrast to exo-acting GHs, ABPs directed to endo-acting ones are perhaps better designed to have the (fluorescent) reporter at the nonreducing end, at the position where the backbone of the natural polysaccharide extents. We took this into account when we designed the set of ABPs **63**-**65** bearing orthogonal fluorophores to characterize the xyloglucan-degrading GH system excreted by the soil saprophyte, *Cellvibrio japonicus*, when grown on various glycan sources (Figure 7B).^72^ Xyloglucanase activity (the red band), which we by chemical proteomics identified to be the Cel5D gene product to which cellulase activity was ascribed, was found to be expressed and excreted only in the samples of *Cellvibrio japonicus* grown on xyloglucan. Growing these bacteria on arabinoxylan yielded an unexpected yellow signal with an apparently unique molecular weight, and if a single protein causes this signal, it must be reactive to both probes **63** and **64**, even though its expression is elicited by a polysaccharide source that does not contain these structural elements.

## Conclusion and Outlook

This year marks the 25th birthday of activity-based protein profiling, with the seminal paper on serine hydrolases^22^ dating back to 1999. Glycosidase ABPP emerged not much later but initially evolved at a much slower pace. This may be because glycosidases are more particular to their substrates, defying the design of a one-size-fits-all ABP. This in contrast to for instance the Cravatt serine hydrolase ABPs^22^ that at once capture hundreds of serine hydrolases. The increasing number of cyclophellitol/cyclophellitol aziridine ABPs becoming available allows for multiplexing ABPP studies to capture multiple retaining GH families at once, and the field is therefore catching up in this aspect. At the same time, they have proven to be high precision instruments that allow for detailed *in situ* and sometimes *in vivo*(73) ABPP studies less easily accomplished by broad-spectrum probes. While at times they show some cross-reactivity toward retaining GHs they were not designed for, nonspecific reactions with other proteins occur only rarely, much less frequent than what is observed when working with ABPs targeting other enzyme families. Retaining GH ABPP is therefore low-tech with targets easily visualized by SDS-PAGE. It is also compatible with high-tech proteomics platforms.

The remarkable selectivity of cyclophellitol-cyclophellitol aziridine ABPs may in part be because these are relatively polar compounds not prone to binding to protein surfaces. As well, they appear not to be very reactive electrophiles. One of the surprising observations we made in synthesizing endoglycosidase probes like **64** and **65**(72) is that one can in fact chemically glycosylate partially protected cyclophellitols/aziridines, thus exposing these functionalities to (Lewis) acidic chemical glycosylation conditions.^74^ Possibly the electron-withdrawing nature of the remaining cyclitol substituents dampen the reactivity of the epoxide/aziridine, at least for S_N_1 substitutions (destabilization of carbocation intermediates, like oxocarbenium ion destabilization in deoxyfluoro glycosides). Once within a retaining GH active site, though, they may benefit from protonation by the general acid/base residue to develop a positive charge which is stabilized by the active site. This may hold true especially for the aziridines, with an estimated (based on the value for the nonsubstituted cyclohexylaziridine) p*K*_a_ of around 8 which is like that of the widely used competitive retaining and inverting GH inhibitors: deoxynojirimycins.

Variation of configuration and substitution pattern has allowed us to capture a range of retaining exo- and endoglycosidases, of either biomedical or biotechnological relevance. We are assembling focused libraries of retaining GH ABPs and have shared our reagents with numerous colleagues who are now using these in their own studies. Redinbo and collaborators apply *glucuronic* cyclophellitol aziridines **54** to map individual gut microbiota retaining β-glucuronidases, both their nature and abundance, and correlates this to intestinal therapeutics (de)activation with the aim to stratify patient populations for drug toxicity/efficacy.^75^ Fleishman and co-workers have used xylobiose aziridine **62** for the identification of potential new xylanase activities, out of a pool of close to one million *in silico*-generated proteins.^76^ Retaining GH ABPs, like ABPs in general, start to prove their worth in (high) throughput settings, which may be for the discovery or engineering of new GH activities but also for the discovery or design of competitive GH inhibitors.^77^ Future research will see more efforts in this direction, as well as in further expansion of the current pool of retaining exo- and endo-GH probes. Retaining neuraminidases for instance employ a tyrosine as nucleophile^7^ which should be reactive toward an appropriately configured cyclophellitol/aziridine as well. Fucoidan comprises a major marine biomass polysaccharide composed of branched fucose backbone highly dotted with sulfate groups, and fucoidan-degrading secretomes contain retaining GHs that can be annotated by approaches as shown in Figure 7B.^6^ Rhamnogalacturonan II, one of our dietary fibers, is a highly complex polysaccharide composed of 21 unique interglycosidic linkages. One single species of gut microbiota, *Bacteroides thetaiotaomicron*, was shown to be able to hydrolyze 20 of these, 13 of which by retaining GHs.^78^ The development of ABPs for such enzymes may require new chemistries, possibly also in the design of the nature of the electrophilic trap. This is an ongoing activity, besides our work most recently for instance in the work^79^ by Andrew Bennet on allylic, carbacyclic glycomimetics.

Inverting GHs have almost completely defied all ABP designs, this while this family is about equal in size as that of the retaining GHs. Brumer and co-workers in their studies on mixed-linkage endoglucanases showed that haloacetyl aminoglycoside-based ABPs may react with the general acid–base residue in retaining GHs (but surprisingly not with the corresponding nucleophile).^80^ Building on this finding, glycomimetic designs that fit in inverting GH active sites to present a suitable electrophile to either of the two active site carboxylates appear feasible. The same holds true for retaining GHs utilizing neighboring group participation, such as the epoxide-forming α-mannanases,^10^ for which we designed spiro-epoxides as a new class of mechanism-based inhibitors and ABPs with promising affinity.^81^ Finally, cyclophellitol-inspired designs may find use in a biomedical context beyond ABPP. Mechanism-based HPSE inhibitor **55** may have potential for the development of new antimetastatic cancer agents.^67^ Cyclophellitol cyclosulfate **57**(68) in turn halts SARS-CoV-2 proliferation in infected lung cells equally effective as the best-in-class N-alkyl-deoxynojirimycins.^82^ This, while **57** selectively inhibits ER α-glucosidase II and does not inactivate the inverting GH, ER α-glucosidase I. These compounds comprise early stage drug candidates, at most. Yet they do demonstrate the potential of mechanism-based inhibitors, beyond serving as blueprints for ABP design, as promising starting points for therapeutics development.
